# Proceedings of the Clinical Microbiology Open 2024: artificial intelligence applications in clinical microbiology

**DOI:** 10.1128/jcm.01804-24

**Published:** 2025-03-27

**Authors:** Jennifer Dien Bard, Andrea M. Prinzi, Paige M.K. Larkin, David R. Peaper, Daniel D. Rhoads

**Affiliations:** 1 Department of Pathology and Laboratory Medicine, Children’s Hospital Los Angeles; Keck School of Medicine, University of Southern California 5116 , Los Angeles, California, USA; 2 US Medical Affairs, bioMérieux, Salt Lake City, USA; 3 American Society for Microbiology 11003 , Washington, District of Columbia, USA; 4 Department of Laboratory Medicine, Yale University 5755 , New Haven, Connecticut, USA; 5 Department of Pathology and Laboratory Medicine, Cleveland Clinic 2569 , Cleveland, USA; 6 Department of Pathology, Cleveland Clinic Lerner College of Medicine, Case Western Reserve University School of Medicine 242911 , Cleveland, Ohio, USA; 7 Infection Biology Program, Lerner Research Institute, Cleveland Clinic 22516 , Cleveland, Ohio, USA; Boston Children's Hospital, Boston, Massachusetts, USA

**Keywords:** artificial intelligence, machine learning, image analysis, clinical microbiology

## Abstract

The Clinical Microbiology Open (CMO) is a meeting sponsored by the American Society for Microbiology (ASM) in collaboration with its Corporate Council and Clinical and Public Health Microbiology representatives, which is held to discuss topics that are relevant to both industry and practicing clinical microbiologists. The 2024 CMO was held in Oceanside, California on February 1 and 2. Participants included clinical and public health laboratory directors, representatives from government agencies, and biotechnology industry partners. The group engaged in discussions with the theme, “The Lab of the Future.” One of the primary topics discussed was artificial intelligence (AI) opportunities in clinical microbiology laboratories. This report summarizes the discussion and sentiment of the group regarding AI tools, opportunities and challenges of AI in clinical laboratories, and potential future directions for AI in clinical microbiology practice.

## INTRODUCTION

ClinMicro Open (CMO) is an annual meeting organized by the American Society for Microbiology (ASM) to foster open dialog and collaboration between the medical microbiology and biotechnology communities. This event serves as a platform for clinical laboratory directors and members of the ASM Corporate Council, comprising biotechnology industry partners engaged in year-long collaborations with ASM, to present their work, network, and discuss the future trajectory of clinical and public health microbiology. The 2024 CMO was held from February 1 and 2 at the Mission Pacific Hotel in Oceanside, California. Participants, including corporate council members, clinical directors, and government representatives, gathered to explore the central theme, “The Lab of the Future.” The first day of the meeting focused on the influence of artificial intelligence (AI) on clinical laboratories, a topic that is the subject of this manuscript.

**Fig 1:**
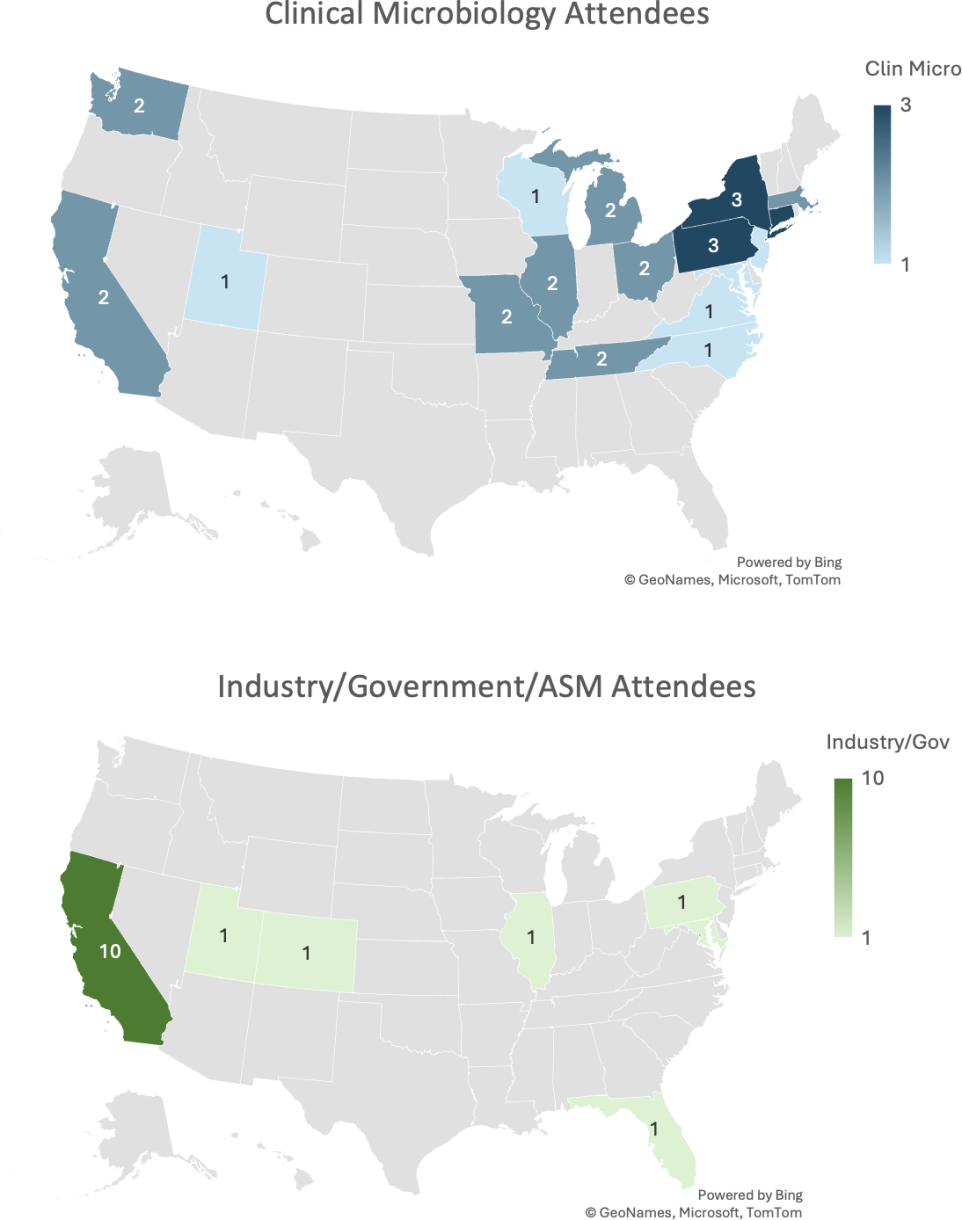
Geographic distribution of CMO attendees. Separated out into clinical microbiology attendees (blue) and industry, government, or ASM attendees (green). Maps created using Microsoft Excel.

Overall, 56 people participated in the CMO event over 2 days. Attendees included clinical microbiologists (director level or higher), representatives of government agencies, ASM staff, and biotechnology industry professionals. Attendance to CMO is a competitive process. A total of 35 clinical microbiologists submitted applications to attend, which included session proposals and statements of interest, with 19 selected by the planning committee for the final event. Industry corporate council members are invited to bring two representatives as a benefit of their corporate council membership. The demographics of the attendees are seen in Table 1, and the geographic representation of attendees is seen in Fig. 1. Table 2 describes the content of sessions presented on day 1, which spanned multiple facets of clinical microbiology including parasitology, Gram staining, plate reading, antimicrobial resistance (AMR) prediction, laboratory efficiency and workflow, and workforce.

**TABLE 1:** Demographics of CMO 2024 attendees

Industry and government *n* = 16 (29%)	No. (%)
Executive leadership (CEO, CFO, CSO, CMO)^a^	4 (25%)
Marketing	3 (19%)
Medical affairs	3 (19%)
Scientific affairs	5 (31%)
Biologist (government)	1 (6%)
ASM Staff *n* = 9 (16%)	
Executive leadership (CEO, CFO, CSO, CMO)	2 (22%)
Advocacy/government affairs	1 (11%)
Operations/program officer/support	4 (44%)
Clinical/public health director	2 (22%)
Clinical Microbiology^b^ *n* = 31 (55%)	
Clinical Microbiology Attendee Institution Demographics	
Reference Lab	4 (13%)
Hospital	27 (87%)
400–600 beds	8 (30%)
601–900 beds	8 (30%)
901–1,400 beds	5 (19%)
>1,400 beds	6 (22%)

^*a*^ Definitions: Chief Executive Officer (CEO), Chief Financial Officer (CFO), Chief Scientific Officer (CSO), Chief Medical Officer (CMO).

^*b*^ Clinical microbiology attendees included director-level microbiologists.

**TABLE 2:** “Lab of the Future” artificial intelligence in clinical microbiology presentations

Presentation topic	Presenter
The Impact of AI on Clinical Labs	Thomas Durant, Yale New Haven Hospital
Use of AI to Interpret Test Results and Quality Monitoring	Meghan W. Starolis, Quest Diagnostics
Use of AI to Detect Parasites	Ben Cahoon, Techcyte
Metasystem AI for Gram Stain Review	Esther Babady, Memorial Sloan Kettering Cancer Center
AI-Based Slide Interpretation: Opportunities and Challenges	Kenneth Smith, Children’s Hospital of Philadelphia
AI and NGS to Predict Antimicrobial Resistance	Romney Humphries, Vanderbilt University Medical Center
Utilizing AI for Culture Plate Interpretations – What Does it Do and What’s Out There?	Susan Sharp, Copan
AI Agar Plate Reading: How Can AI Optimize Laboratory Efficiency?	Erin McElvania, Northshore University Health System
How Can AI (and Automation) Improve Our Workforce?	Laura Filkins, University of Texas Southwestern Medical Center

## DEFINITIONS OF AI TOOLS

In the simplest form, AI is defined as having machines perform tasks that would typically be performed by humans using trained intelligence. AI is a powerful computational tool that has gained traction across all facets of medicine, including laboratory medicine. AI has been showcased in blockbuster movies and television shows for decades, and some of the potential of AI is finally coming to fruition in the real world. For the practice of medicine, the ability of AI to carry out human functions with enhanced speed and diagnostic accuracy is certainly appealing. Advancements in AI and machine learning (ML) have been particularly evident within the specialty of radiology to screen multi-disciplinary imaging sets to detect, measure, classify, and monitor malignancies (1). Table 3 provides definitions for key terms within AI.

**TABLE 3:** Definitions of terms in artificial intelligence

Terminology	Definition
Artificial intelligence (AI)	A branch of computer science. AI systems use hardware, algorithms, and data to create “intelligence” to do things like make decisions, discover patterns, and perform actions.
Algorithm	The “brain” of the AI system, an algorithm is a set of rules for what action the AI system takes. This may include rules that the algorithm may be trained to discover or rules that are built by human programmers.
Superintelligent AI	A theoretical system that is more intelligent than humans in cognitive, memory, reasoning, and learning functionality.
Artificial narrow intelligence (ANI)	AI that is designed to perform a single or narrow set of tasks.
Neural networks	This subset of machine learning algorithms uses deep learning to teach computers to process data in a way that mimics the neurons and synapses of the human brain.
Deep learning	A subset of machine learning that uses multilayered neural networks to simulate the complex decision-making of the human brain. Unlike non-deep machine learning, which uses simple neural networks with 1–2 computational layers, this approach uses neural networks with hundreds or thousands of layers to train the model.
Training data	The data used to train the algorithm or machine learning model. It is important to note that algorithms take on the biases that exist within the training data.
Black boxes	A term used to describe the algorithms in which we do not have an understanding of how the system is using features of data to make decisions.
Machine learning	A type of AI that identifies rules and patterns in data without a human specifying them. The algorithm discovers its own rules in the data it is provided.
Expert systems	A computer program that uses AI to simulate human expertise or judgement in a specific field. These programs are intended to aid decision-makers, not replace them.
Adaptive AI	Any type of AI that learns and adapts to new data and environments. In theory, adaptive AI should improve over time, adjust appropriately to changes, and make faster decisions.

The applications of AI can be categorized into three types based on their capability. Narrow AI is designed to perform designated tasks or solve specific problems, such as speech recognition or image classification. This is currently the only form of AI available, and it has been adopted in laboratory medicine. The remaining two categories, General AI (a hypothetical concept that is capable of understanding, learning, and applying reason like humans) and Superintelligent AI (a theoretical concept that is capable of surpassing human intelligence and outperforms humans), currently do not exist and are subjects of much ethical debate (2).

Narrow AI includes classical programming, which functions around rules and algorithms defined by humans. Expert systems, which are computer programs that leverage AI to support humans in decision-making, are a prime example of Narrow AI as they depend on human-curated knowledge and rules (3). Applications within clinical microbiology diagnostics that depend on expert systems include commercial organism identification and antimicrobial susceptibility test (AST) systems (e.g., Vitek2, BD Phoenix). Molecular technologies commonly used (e.g., GeneXpert systems, BioFire FilmArray) within clinical microbiology laboratories rely on rule-based programming to accurately detect the targets of interest. These systems often include a knowledge base comprised of domain-specific knowledge that is used in conjunction with rules or algorithms to deduce new information, make inferences, or solve problems. A primary limitation of the rules-based approach is the absence of learning or adaptability.

In contrast to classical programming, adaptive AI evolves by processing vast amounts of data through ML or data-based algorithms to improve performance without being explicitly programmed for every scenario. The three main types of ML tasks are classification (predict categorical outcomes), regression (predict continuous values), and clustering (group data based on similarities). The two common types of data used within the clinical microbiology laboratory are tabular data and image data. Tabular data refer to how data are organized in table form, typically in rows and columns, and often forms the backbone of ML models within the clinical microbiology laboratory. Examples of tabular data include the use of classification algorithms to categorize bacteria as susceptible or resistant to a particular antibiotic, regression algorithms to predict the minimum inhibitory concentration (MIC), and clustering algorithms to group bacteria with similar resistance patterns together. As the interpretation of microscopic and macroscopic images is fundamental to clinical microbiology, adaptive AI using image data classification and clustering to recognize organisms and group them together based on visual similarities is highly appealing (4, 5).

## ESTABLISHED AND EMERGENT AI TECHNOLOGIES IN THE CLINICAL LABORATORY

AI, specifically narrow AI, has had a steady yet rather silent presence in the clinical microbiology laboratory for decades. In recent years, with the emergence of exciting adaptive AI tools, the exploratory potential within clinical microbiology diagnostics appears endless.

### Image analysis artificial intelligence

Image analysis, otherwise known as computer vision, is an intriguing AI approach that detects and interprets microorganisms in the clinical laboratory (6). The development of advanced, multi-layered AI architectures, classified as deep learning algorithms, has enabled the interpretation of raw digital images by comparing them to carefully annotated reference standards. These algorithms fall under the broader category of ML where systems can adjust and learn from data independent of being programmed. For instance, supervised ML is a subset of MI that uses a set of annotated images as its reference standard to develop a complex algorithm to interpret unprocessed data (5). A common type of supervised ML algorithm used for image analysis is convolutional neural networks (CNNs), which are modeled after the human visual cortex and employ highly interconnected networks. By analogy to human intelligence that learns from experience, CNNs adjust their internal parameters when stimulated by input images, allowing them to recognize and classify patterns effectively (7). The three stages required for the development of a supervised ML algorithm such as CNN are: training, validation, and testing (5). The training stage cannot be underscored, as the robustness and accuracy of the algorithms are dependent on the quality of the material available. An article by Smith and Kirby nicely summarizes the intricacies of image analysis using CNNs (7).

Image analysis AI using CNNs has been adopted throughout many medical specialties. Within pathology, image analysis AI approaches have been incorporated into histology, cervical cytology, and hematology applications. Specifically, CellaVision DM9600, an automated device and blood differential software, has proven to be a robust AI tool in detecting, identifying, and pre-classifying white blood cells and red blood cells for a differential (8, 9). The approach has also been explored for its capability to detect parasitic inclusions within red blood cells (10, 11).

### Applications of image analysis AI in clinical microbiology

The transformative potential of image analysis AI in clinical microbiology diagnostics has been increasingly recognized and explored for organism identification on agar plates, ova and parasite detection, and blood parasite detection. Established applications in the clinical laboratory include the use of ML algorithms for bacterial colony detection and bacterial culture interpretation. For example, ML software has been paired with chromogenic media to recognize the morphological characteristics of the organism and provide an identification. Studies have reported highly accurate classification of organisms such as vancomycin-resistant enterococci (VRE), methicillin-resistant *Staphylococcus aureus* (MRSA), and *Streptococcus pyogenes* (12–14) on specific chromogenic media. Nonchromogenic solutions to bacterial culture interpretation have also been explored including the classification of urine culture plates based on colony counts (15). Image analysis software can also aid in AST interpretation such as measurement of disk diffusion zones of inhibition and interpretation of broth microdilution growth patterns to determine MICs (16–18).

Leveraging image-based ML for microscopic images is particularly of interest in the clinical microbiology laboratory. Validation of a CNN model to analyze digital microscopic images and accurately detect protozoa in trichome-stained fecal smears has recently been explored (Techcyte Inc., Linden, UT) (19). Mathison et al. conducted extensive training between 1,394 and 23,566 exemplars per class (various targeted species and morphological stages of protozoa) as part of the ML algorithm and reported a robust tool for augmenting the conventional detection of intestinal protozoa with a positive and negative percent agreement of greater than 98% (19). Image analysis AI has also been explored for the detection and identification of blood parasites directly from digital images of blood smears with promising preliminary findings. A recent study assessed the performance of a fully automated malaria diagnostic system (EasyScan GO, Motic Digital Pathology) and reported a detection accuracy of 94.3%. However, species-level identification was 82.9%, and quantification accuracy was 50% in this system, highlighting the complexity of these ML applications and the importance of the three stages of development previously mentioned (20).

Gram-stained slide review remains one of the mainstays of clinical microbiology diagnostics; thus, the potential to automate the review and interpretation of Gram-stained slides through AI tools has the potential to make a large positive impact on operational efficiency. Recent studies evaluating Gram-stained slides from positive blood cultures, respiratory samples, and other sterile fluids using a combined fully automated digital microscopy with CNN model to assist the interpretation of Gram stains (MetaFer Slide Scanning and Imaging platform; MetaSystems Group, Inc. Newton, MA) revealed comparable results with a manual review (21, 22). Walter et al. conducted a study of 1,555 Gram-stained slides from blood cultures positive for gram-positive bacteria, yeast, and polymicrobial specimens. The positive percent agreement (PPA) for gram-positive cocci in clusters and rod-shaped bacilli was greater than 97%, whereas the PPAs for gram-positive cocci in pairs/chains and yeast were 87.6% and 83.3%, respectively (23). Overall, the studies reveal clear potential for image analysis AI for Gram stain review. An interesting approach to AI and Gram-stained smears is to screen for bacterial vaginosis. A CNN model has recently been developed and optimized to classify Nugent scores on digitized vaginal smears (24). The researchers reported that the deep learning model outperformed the medical technologists reviewing the Gram-stained slides, with an increase in accuracy of 6.6% (24).

Finally, an innovative approach to Gram-stained slide interpretation is the application of digital holographic microscopy (DHM), which captures interference patterns created by laser light interacting with a sample and the computational determination of morphological and physical parameters of living cells, in the absence of objectives and manual adjustments. The resulting image is equivalent in clarity and detail to what would be seen under a traditional microscope, but the process is much faster and more precise. As DHM identifies altered cell morphologies, it serves as a promising tool for microorganism characterization (25).

Beyond the culture-dependent and microscopic-based image analysis AI applications, AI may offer solutions to improve rapid identification and AST, AMR detection, and molecular interpretation including bioinformatic analysis of next-generation sequencing (NGS) data. Advanced AI techniques, such as graph-based machine learning (GML) that analyze and leverage the relationships between data points, are also being explored to understand complex relationships between pathogens, genetic data, and AMR profiles, potentially offering deeper insights into AMR mechanisms (26, 27).

## BENEFITS OF AI IN THE CLINICAL LABORATORY

### Laboratory operational improvements

The integration of AI, particularly ML, in clinical microbiology laboratories offers significant advantages in streamlining workflows. A prime example is in molecular microbiology laboratories that utilize Polymerase Chain Reaction (PCR) methods. Data analysis and quality control (QC) review remains a manual and burdensome process for medical laboratory scientists. Manual review and interpretation are required for laboratory-developed tests (LDTs) as well as many commercial PCR assays (28). Review of amplification curves is not intuitive and can be challenging even for well-trained laboratory staff, and thus, interpretation can be time-consuming and prone to interpretation error.

Automation of manual review through the implementation of AI tools can enhance efficiency and accuracy (29). ML algorithms can be trained using historical data to analyze quality parameters, review PCR hybridization curves, and enforce QC rules. By leveraging previous data, ML can identify and train on “appropriate” PCR curves using kinetic parameters, allowing users to review and adjust calibrations as needed to ensure accuracy. The use of “if-then” logic, currently used for hybridization curves, can verify other QC parameters including contamination or carryover, internal control value, and acceptability of controls under pre-defined criteria. Novel and emerging AI solutions could fundamentally change the approach to quality monitoring systems in the clinical microbiology laboratory (30). Benefits of these ML tools include reducing human error and subjectivity, enhancing data analysis and rigorous quality standards, and expediting the analysis process. The PCR.Ai (59a Brent Street, London, UK) is an automated tool that utilizes ML as well as if-then logic to automate the post-analysis processes for PCR tests. A study from the UK compared the PCR.Ai system against manual interpretation of PCR assays for a total of 22,200 interpretations and reported 100% concordance. The authors also reported average time savings of 40 min per run and estimated an annual savings of 160 h based on one run per day over a 5-day week (28). Furthermore, a recent study explored PCR.Ai for qualitative PCR assays (CMV, EBV, and adenovirus) and confirmed 100% concordance with an estimated time savings per run at 63 min per run, which would be expected as quantitative PCR assays require more complex interpretation (31).

The implementation of bacteriology automation and digitization (often termed “total laboratory automation” [TLA]) in the clinical microbiology laboratory has led to levels of standardization and efficiencies in both pre-analytic steps (e.g., specimen processing, plate streaking, and incubation) as well as through early growth detection, better culture quality, and shorter turn-around times (32). Leveraging the availability of digitized images as part of TLA alongside ML algorithms can further improve efficiency. For instance, AI tools can facilitate the sorting and prioritization of cultures for review, streamlining the process of identifying organisms. By implementing dynamic culture reading worklists, AI tools can organize the workflow based on various criteria, including prioritizing based on specimen types, organism burden, and patterns including monomicrobial or polymicrobial growth. Algorithms can also be developed to flag unusual or suspicious results for MLS review (e.g., plate contaminants, unusual growth trends for a specific lab, or correlation of Gram stain and culture results). This prioritization enables laboratory personnel to focus on the most critical specimens first, streamlining the culture work-up process. ML can also be used to train software to interpret culture growth like a human interprets it (33). In the context of disk diffusion AST, AI algorithms can provide early susceptibility information, streamlining organism-specific reading times and interpretation.

The use of AI in the interpretation of culture results would further improve laboratory efficiency. A recent study summarized the development of *DeepColony*, a hierarchical multi-network application capable of handling all identification, quantitation, and interpretation stages. When compared with interpretation of MLSs, *DeepColony* was also able to confirm negative culture results with >99% agreement and positive cultures with >95% agreement. The authors noted that *DeepColony* was able to effectively classify and enumerate organisms that can be paired with laboratory-based rule systems to aid in the interpretation of clinically significant cultures (34).

Development and implementation of robust AI tools for culture review and interpretation can significantly enhance access to microbiological diagnostics in rural and community hospitals, where resources and specialized personnel may be limited. Furthermore, the implementation of digitized images and AI-driven systems allows for remote consultations from larger reference laboratories, facilitating expert review of complex cases.

### Combating AMR and outbreaks

### How can AI help fight AMR?

AMR remains one of the biggest threats to modern medicine, and significant efforts are being made to improve AST methods in the clinical laboratory, including faster phenotypic and genotypic AST approaches. AI is poised to play a pivotal role in the fight against AMR by leveraging advanced computational techniques that allow for the analysis of large data sets, enabling key functions like data analysis and pattern recognition. This involves examining bacterial genomic data, patient records, and environmental factors to identify trends and emerging resistance. ML models can also be used for predictive modeling, aiding clinical decision support and rapid diagnosis by providing real-time, data-driven insights on pathogen identification and antibiotic efficacy. Additionally, AI systems support continuous learning, helping prevent AMR and guiding the development of new antibiotics by integrating the latest data and evolving trends.

Whole genome sequencing (WGS) methods are increasingly used for epidemiological investigations, and they have more recently been explored for resistance profile predictions by pairing AMR genes to phenotypic AST results. This “look-up table” model requires well-curated and developed databases of genes and mutations, but performance can vary based on bug/drug combinations. For instance, this approach can effectively predict antimicrobial resistance in such organisms as *S. aureus, Mycobacterium tuberculosis* complex, and HIV and is a promising alternative to conventional approaches (35–37). However, there may be limited clinical utility for some groups, such as gram-negative organisms, due to poor correlation between AMR genes and phenotypic AST results (38).

The alternate genomic analysis approach that has been explored with WGS is the ML approach, which can analyze multiple genomic features to predict phenotypes more accurately. To ensure accurate AST predictions, deep learning techniques would be trained against a robust data set comprising thousands of genomic data labeled as either susceptible or resistant to the antibiotics of interest. Ideally, the determination of these labels can be influenced by various factors, including the breakpoint criteria, the source of the organism, and the timing of its isolation. AI tools have demonstrated promising performance. Preliminary data evaluating the performance of the Next Gen Diagnostics (NGD) ML pipeline to predict cefepime susceptibility on a cohort of 100 *Escherichia coli* urine and blood isolates reported a higher categorical agreement (CA) (97%) compared with the reference BMD method (95%) (39). A previous study reported the development of an extreme gradient boosting (XGBoost) based ML model to predict MICs for 15 antibiotics using WGS and AST data from 5,278 nontyphoidal *Salmonella* strains. The model of using k-mers to predict both resistant and susceptible MICs was robust with a mean accuracy of 95% (40). Certainly, the data summarized are promising and encourage wider development of sequence-based susceptibility prediction. Exploring faster analytical and post-analytical applications will further increase the clinical relevance of such explorations (41). Refer to a recent review article by Pérez de la Lastra et al. for additional studies exploring the role of AI and ML in AMR detection (42).

From the microbiologist and clinician’s perspective, it would be ideal to build these ML models so that they are not black boxes, but instead, data can be dissected to reveal biologically plausible causal genomic elements. The overarching goal would be the creation of an ML model that could also predict other *in vivo* measurable phenotypes from WGS, such as the likelihood of persistence, recurrence under treatment, or 30-day mortality.

#### Outbreak and reservoir detection in the hospital setting

Using ML tools to incorporate genomic drivers of resistance and improve clinical microbiology testing is appealing. Furthermore, the integration of electronic medical record (EMR) data with ML models could help predict the presence and timing of risk factors for adverse patient outcomes or nascent nosocomial outbreaks. This would allow interventions to be made to potentially avoid these outcomes. These ML tools can also inform antibiotic stewardship programs (ASPs) and infection prevention and control strategies, ensuring that healthcare providers can make informed decisions that optimize patient care and resource allocation. The role of WGS and ML tools have been well explored for outbreak investigations and have proven to be valuable for identification of the occurrence of outbreaks, potential sources or routes of transmission, and implementing infection prevention and control practices (43, 44). Specifically, findings from integrated genomic-epidemiologic analyses of an outbreak of multi-drug-resistant *Shigella* among men who have sex with men (MSM) were leveraged to direct targeted clinical actions to improve rapid diagnosis and patient care and for public health efforts to further reduce spread (44).

An example of how AI tools can be leveraged is for predicting the risk of *Clostridiodes difficile* infections. Asymptomatic colonization of *C. difficile* poses a significant risk for infection; however, the lack of comprehensive surveillance data leaves healthcare providers operating in the dark. Employing AI-driven approaches can enhance cluster detection, identify transmission drivers, and pinpoint where and how to intervene. A recent study of 1,897 ICU patients revealed that 7.4% of patients asymptomatically carried *C. difficile.* Analysis of the genomic and epidemiologic data from the EMR revealed that the relative risk for developing active infection from asymptomatic carriage of a toxin-encoding strain was 9.32. Interestingly, the risk of developing active infection was also associated with increased hospital lengths of stay, re-admission, and mortality (by 67%) in carriers when compared with mortality rates in noncolonized ICU patients (45).

### Combat workforce challenges

Another beneficial byproduct of incorporating AI tools into the clinical laboratory workflow is it serves as a promising solution to combat workforce challenges. Leber and colleagues previously highlighted the current clinical microbiology staffing conundrum that has been further exacerbated by difficulties in MLS recruitment, training, and retention. These barriers unfortunately coincide with testing volume increases, laboratory consolidations, and increased testing complexity. Clinical microbiology in particular is a highly complex laboratory specialty, and the length of time required to ensure proficiency and expertise can be up to 1 year (46). Thus, improved operational efficiencies that were described above will directly target the workforce challenges.

Replacement of time-consuming, repetitive, and sometimes mundane tasks with AI approaches can re-direct MLSs to other more complex tasks but can also improve work satisfaction and retention. This would also allow for absorption of the increase in testing volume in the absence of additional staffing. As digitization and image analysis AI become increasingly integrated within the clinical laboratory to triage culture workup and provide interpretation (4), remote work is possible, offering numerous advantages to employees. A positive byproduct for the laboratory and institution is the reduction in required laboratory space if a portion of the workforce is remote.

### Improving the time to publication and review of manuscripts

Although not directly tied to laboratory diagnostic work-up, the use of AI tools to aid in the advancement of knowledge sharing is increasingly common (47). Publication of key diagnostic advancements and robust data serves as a powerful tool of communication within the scientific community, fostering the continual expansion of knowledge and learning. There have been rapid advances in generative AI that can create new text output, images, audio, and video that mimic products that would be created by humans. This includes leveraging generative AI Large Language Models (LLMs) to accelerate productivity by summarizing and drafting papers. Well-known examples of LLMs include tools like ChatGPT and Google Bard. Researchers are increasingly turning to generative AI to assist in writing, reviewing, and creating graphics for their manuscripts, in an attempt to enhance both the efficiency and quality of scientific communication. For researchers who may not have English as their first language, these AI tools can provide invaluable support through editing and translation.

## CHALLENGES OF AI IN THE CLINICAL LABORATORY

### Challenges of AI for agar plate reading and AST

The integration of AI and automation for plate reading in clinical microbiology laboratories presents several significant challenges. Implementation requires the development of new protocols and workflows to optimize operational efficiency, which can be particularly complex given the diversity of laboratory settings. The heterogeneity of clinical microbiology practice, especially with bacterial culture, is also a potential barrier to adopting an interpretive algorithm that is more subtle than a straightforward positive/negative result. Each laboratory typically has its own rules based on specimen source, mixture of organisms, and quantity of growth, which determine when an isolate should or should not be identified and tested for susceptibility. Moreover, in clinical microbiology, plate reading often depends on the ability to assess individual bacterial colonies, quantify their growth, and differentiate between various colony types (7). Variations in colony morphology based on bacterial density and media type pose further computational challenges for AI systems. As a result, AI may be more effective for plates with no growth than for those with mixed or complex growth patterns, where it may struggle to distinguish between different colony types. The development of effective AI systems for microbiology must also consider training data quality. Human interpretation of culture growth often produces inconsistent data, which is not optimal training material for ML. Instead, meticulous attention to the curation of well-annotated and accurately defined training sets is often preferred (48). Poorly curated training sets can lead to suboptimal AI performance and may perpetuate errors if inadequate practices are inadvertently learned. This risk underscores the importance of carefully defining training and test sets to ensure they are properly segregated and validated.

To advance the use of AI in clinical microbiology plate reading, a collaborative approach that combines laboratory expertise with commercial vendor support is essential for the development of robust AI algorithms. Drawing lessons from other fields, such as radiology, suggests that the highest accuracy in AI applications is achieved when gold-standard training set labels, such as colony counts or binary growth interpretations, are based on multi-reviewer expert consensus standards (48). Such approaches could significantly enhance the reliability and accuracy of AI systems for plate reading.

Although the use of AI for AST and AMR prediction has shown great promise, there are several challenges that currently limit its use. ML models have been shown to provide accurate predictions for AST when trained on large foundational data sets, but these are limited in number due to their size, particularly for public databases, and the cost of adding new sequences (PMID 33536291). To overcome time and data paucity challenges, transfer machine learning has been used increasingly. Transfer ML is the process of using the knowledge gained from one data set to inform a new model (PMID 36421255, PMID 38138930). It is possible that an ML algorithm will identify an AR determinant in the training set that is specific in some way (i.e., a certain part of the world). This feature may not be predictive or representative when used for a different population or part of the world. Without knowing what the ML model is keying in on in the training set, it is impossible to predict transferability, and generalizability may be limited. Finally, significant variability across inputs poses a significant challenge to the widespread adoption of AI for AST. The variations in AST methods, breakpoints, EHR data (i.e., structured and unstructured, entry error, different databases, and nonrandom missing observations), and differences in practice across institutions should all be considered and addressed as thoroughly as possible, ensuring attention to local context and needs (PMID 33536291).

Another critical consideration for the integration of AI across microbiology methodologies is the distinction between near-patient testing facilities and centralized laboratories. Large, centralized laboratories are more likely to adopt automation due to economies of scale, whereas the high costs associated with total automation and associated AI technologies make it less feasible for deployment in multiple decentralized locations. Currently, the capacity to develop and maintain AI systems is beyond the capabilities of most clinical laboratories, necessitating a reliance on commercial vendors for support. This dependency brings additional regulatory complexities, and significant time and expertise are required to address AI malfunctions or failures. Currently, plate reading and AST are primarily performed at a core laboratory location where the integration of AI is more feasible. However, as technologies advance, and the possibility of bringing these methodologies closer to the point of care emerge, this is a point that will require extensive consideration.

### Challenges of using AI to publish or review manuscripts

A study by Richard Van Noorden and Jeffrey M. Perkel, which surveyed 1,600 researchers, highlights several key concerns about the use of generative AI (GAI) and large language models (LLMs) in scientific research and publishing (47). The potential issues identified include the proliferation of misinformation, increased ease of plagiarism and difficulty detecting it, the inadvertent introduction of errors or inaccuracies into research texts, the facilitation of research fabrication, and the risk of entrenching biases or inequities in scientific literature. These challenges raise fundamental questions about the appropriate use of GAI and LLMs in scientific authorship and peer review. One major point of contention is whether GAI or LLMs can be credited as authors on scientific papers. According to the criteria set by the International Committee of Medical Journal Editors (ICMJE), authorship requires: (i) substantial contributions to the conception, design, acquisition, analysis, or interpretation of data; (ii) drafting the work or revising it critically for intellectual content; (iii) final approval of the version to be published; and (iv) agreement to be accountable for all aspects of the work, ensuring its accuracy and integrity (49). Given that GAI and LLMs cannot provide final approval or accountability, both the ICMJE and ASM have concluded that these tools cannot be considered authors. The ICMJE explicitly states, “Chatbots… should not be listed as authors because they cannot be responsible for the accuracy, integrity, and originality of the work (49),” whereas the ASM journals policy notes, “AI tools cannot be considered authors of ASM journal articles, as they do not have agency, cannot be said to provide intellectual contribution, and cannot sign or agree to the terms of copyright transfer (50).”

Despite these restrictions, the use of GAI and LLMs in manuscript preparation is permitted by the ICMJE and most publishers, provided that their use is fully disclosed, and human authors take full responsibility for the content’s accuracy, references, and originality. Initially, some journals, such as those in the Science family, banned the use of GAI and LLMs on the grounds that text generated by these tools would not be considered “original,” as authors are required to certify the originality of their work. However, these journals have since revised their policies to permit the use of AI-generated text, albeit with the requirement of editorial permission and transparency. For instance, the creation of AI-generated images necessitates explicit editorial approval.

At CMO 2024, a case study of detecting the use of GAI in a manuscript was presented, highlighting several significant concerns associated with its use in scientific publishing, including the tendency to generate “tortured phrases” (e.g., "mixed drinks" instead of “cocktails” when referencing antibody cocktails), inaccuracies in referencing, and nonsensical sentences, which can undermine the integrity of scientific literature. Moreover, the use of these tools in peer review raises additional ethical concerns. The National Institutes of Health (NIH) prohibits the use of GAI and LLMs in the peer review of grants due to the risk of confidentiality breaches, as these tools may retain and reuse submitted text for further training (51). This risk is a primary reason many publishers also restrict the use of GAI and LLMs in the peer review process, as they could inadvertently compromise the confidentiality and integrity of sensitive review materials.

Mitigating these risks may involve exploring alternative approaches, such as deploying privately hosted AI systems that do not retain user data. However, the extent to which these measures address concerns about confidentiality, especially in cases where preprints are involved, remains an open question. The application of GAI and LLMs in scientific and clinical publications is an evolving and expanding field. Authors, editors, and publishers are continuously innovating and experimenting with these technologies, which offer unprecedented capabilities for analyzing and generating text. However, these advancements challenge long-established publication practices and raise concerns about the accuracy of content and ethical standards. It is essential to address these concerns to fully harness the utility and potential of GAI and LLMs in improving and democratizing the publication industry.

## CONSIDERATIONS FOR AI USE IN THE FUTURE

### Using AI and automation to improve the medical laboratory workforce

The medical laboratory workforce has been experiencing a persistent crisis, characterized by a significant mismatch between job vacancies and the number of medical laboratory science (MLS) training program graduates, high rates of retirement, and decreasing rates of job satisfaction. This situation was exacerbated by the COVID-19 pandemic, which underscored the critical need for trained medical laboratory professionals and highlighted the consequences of inadequate support for this workforce. The underlying causes of this workforce shortage are multifaceted. Key contributing factors include the increasing rate of retirements, relatively lower salaries compared with other healthcare professions, competition with non-laboratory employers, and limited opportunities for career advancement within the field (46). The pandemic-induced shift toward remote work has become a lasting change for many professionals across various industries. Current estimates indicate that approximately 13% of full-time employees in the United States are now fully remote, with an additional 28% working in a hybrid model (52). Notably, individuals aged 24–35 are reported to value remote work highly, with 98% of workers of all ages expressing a desire to work remotely at least part of the time. Although remote work opportunities for MLS are presently limited, the healthcare sector ranks fourth among industries with potential for remote work, suggesting a foundation for future expansion in this area.

A fully automated laboratory challenges the conventional notion that physical presence in a clinical laboratory is essential for completing laboratory tasks. Many stages of the laboratory workflow could potentially be performed remotely. A study by Culbreath et al. demonstrated the positive impact of TLA across four laboratories, highlighting improvements in workflow efficiency, staff utilization, and turnaround times (53). Various steps in the specimen processing workflow could be augmented by laboratory automation and AI, potentially reducing the number of MLS required on-site. For instance, an automated Gram stainer could be integrated with AI-based Gram stain interpretation, allowing for the remote selection of colonies for culture reading. Pure colonies necessitating further testing could be processed using automated technologies such as Matrix-assisted laser desorption ionization-time of flight mass spectrometry (MALDI-TOF MS) target spotting and AST, all facilitated through remote interfaces. Although certain tasks, such as add-on testing and sub-culturing, would still require in-person work, a substantial portion of laboratory work could be conducted remotely, thereby alleviating staffing pressures within the laboratory. The feasibility of remote laboratory work is further supported by existing regulatory frameworks. The CLIA ’88 post-public health emergency guidance document permits pathologists and other laboratory personnel to review digital laboratory data, results, and images remotely without requiring a separate CLIA certificate for the remote site (54). This guidance considers digital data accessed via VPN or other secure methods as an extension of the laboratory, thereby eliminating the need for on-site equipment such as microscopes.

However, several challenges must be addressed to fully leverage laboratory automation and AI in supporting the workforce. These include the need to redesign laboratory spaces to accommodate AI and remote work capabilities, ensure consistent and seamless connectivity, rethink consultation and personnel oversight models, and develop software and contracts that support multiple users in a remote environment. Additionally, existing laboratory workflows and procedures may need to be entirely restructured to integrate these new technologies effectively. Despite these challenges, the potential benefits of implementing remote work in the medical laboratory setting are significant. These include the creation of “clean” space reading rooms, increased job satisfaction among staff, and a reduction in the physical space requirements of laboratories. In light of the ongoing decline in resources and support for the laboratory workforce, exploring the integration of remote work through automation and AI may be a critical step toward ensuring the future viability of the microbiology laboratory.

## FUTURE REGULATORY CONSIDERATIONS FOR AI

There are numerous uncertainties regarding the regulatory management of AI technologies, particularly in comparison to other diagnostic tests currently approved by the Food and Drug Administration (FDA). In 2023, the FDA released guidance recommending the inclusion of a Predetermined Change Control Plan (PCCP) in submissions for AI and machine learning (AI/ML) devices (55). This guidance seeks to streamline the process by which manufacturers update their AI/ML devices while maintaining regulatory oversight. Within the PCCP, the manufacturers are required to pre-specify anticipated changes to the AI/ML software, such as algorithm modifications or retraining with new data. The modification protocol should outline the methods and procedures for implementing these changes, which may involve data management, software validation, and performance testing. Additionally, manufacturers must assess the potential impact of these changes on device performance, supported by scientific evidence demonstrating that the device’s safety and effectiveness will remain intact post-modification. If both the 510(k) submission and PCCP are approved, manufacturers are permitted to implement the pre-specified changes without requiring additional FDA approval, provided all conditions outlined in the PCCP are adhered to.

In a collaborative publication from various FDA centers, including the Center for Biologics Evaluation and Research (CBER), Center for Drug Evaluation and Research (CDER), Center for Devices and Radiological Health (CDRH), and Office of Combination Products (OCP), the FDA outlined its approach to the development and regulation of AI technologies across the medical product lifecycle (56). Key strategies include the following:

- Collaborating with developers, patient advocacy groups, academia, global regulators, and other stakeholders to establish a regulatory framework that prioritizes patient safety and equity.
- Monitoring trends to identify gaps and opportunities for the integration of AI in the medical product lifecycle, supporting regulatory science to evaluate AI algorithms, and understanding their resilience to changing inputs and conditions. This includes issuing guidance on these areas of focus.
- Developing and refining criteria for the safe, ethical, and responsible use of AI in healthcare. The FDA aims to promote best practices for real-world performance monitoring, which may include ensuring the data used to train and validate AI models is fit for purpose and creating frameworks for continuous monitoring and risk mitigation.

Despite these developments, several questions remain within the clinical microbiology community. In particular, there is uncertainty regarding how functional effectiveness will be managed between device clearances and subsequent updates, and by whom. Recent challenges, such as those related to the FDA’s LDT rule and AST breakpoint updates (57), have underscored the complexities of the diagnostic regulatory landscape. Additionally, the roles of state and federal regulatory bodies in overseeing the integration of AI/ML into clinical diagnostics and public health remain unclear.

## CLINICAL MICROBIOLOGY OPEN AUDIENCE SURVEY RESULTS

At the end of the AI-themed session at the 2024 CMO, audience members were asked to complete a short five-question survey primarily focused on their viewpoints on AI development and implementation in the clinical microbiology laboratory. All clinical microbiology and industry attendees were invited to participate, as appropriate, since some questions were relevant for both groups. It is important to note that the overall survey sample size was small, given the number of attendees, with the maximum number of respondents reaching 38 people. The data (Table 4) reflect a growing optimism about the role of AI in clinical microbiology, with 62% (*n* = 23) of respondents feeling that AI is underrated compared with their interpretation of the general public’s viewpoint. This suggests that clinical laboratorians and their industry partners are more hopeful about AI’s potential to enhance patient care compared with their assumption of the general public’s stance. Specifically, microbiology professionals see significant opportunities for AI to streamline operations through the improvement of efficiency of testing (49%, *n* = 18) and enhancing quality monitoring (25%, *n* = 9). Testing accuracy and improved revenue were deemed to be the least beneficial aspects of AI at a combined 13% (*n* = 5).

**TABLE 4:** Summary of post-session open audience survey: Will you use AI in the future and how?

Question	Survey options and participant responses N (%)				
What area are you most optimistic that there will be a near-term AI solutions that could substantially improve clinical microbiology practice? (*N* = 38)	Quality monitoring 8 (22)	Routine interpretation of microscopic images 12 (31)	Routine interpretation of bacteriology culture plates 9 (25)	DNA sequencing interpretation 5 (15)	Tools to provide customized recommendations to caregivers collecting specimens and/or prescribing antibiotics 4 (7)
When do you expect to begin to routinely use AI tools in your clinical microbiology laboratory to support quality monitoring (*N* = 34)	My laboratory already uses AI for this application 3 (8)	2024 or 2025 11 (32)	2026 or 2027 6 (19)	After 2027 12 (35)	Never 2 (5)
When do you expect to begin routine AI use of image analysis tools in your clinical microbiology laboratory to support the standard of care? (*N* = 32)	My laboratory already uses AI for this application 6 (17)	2024 or 2025 10 (31)	2026 or 2027 7 (23)	After 2027 9 (29)	Never (0%)
What do you expect to be the most beneficial aspect of the adoption of AI tools in clinical microbiology? (*N* = 37)	Improved test accuracy 3 (9)	Improved efficiency of testing 18 (49)	Improved turnaround time 5 (14)	Improved quality monitoring 9 (25)	Improved revenue 2 (4)
How do your feelings about the opportunity for AI to positively impact patient care in general compare to the public’s general feelings toward AI? (*N* = 37)	I feel AI’s potential impact is overrated 11 (30)	I feel AI’s potential impact is underrated 23 (62)	I feel AI’s potential impact is similar to how the public feels 3 (8)		

When considering the area that the respondents were most optimistic to be near-term AI solution that could substantially improve clinical micro practice, image analysis AI tools such as routine interpretation of microscopic images (31%, *n* = 12) and bacteriology culture interpretation (25%, *n* = 9) received the highest scores. Respondents do see a near-term AI solution for quality monitoring (22%, *n* = 8) as well but were less optimistic about more generative AI Tools to provide a customized recommendation to caregivers collecting specimens and/or prescribing antibiotics (7%, *n* = 2).

Regarding the timeline for AI adoption in clinical microbiology laboratories, only 17% (*n* = 5) and 8% (*n* = 2) of laboratories are currently using AI tools for image analysis and quality monitoring, respectively. An additional 31% (*n* = 9) expect to adopt image analysis AI by 2024 or 2025, which aligns with expectations for AI-driven quality monitoring, where 32% (*n* = 10) of labs anticipate using any form of AI within the same timeframe. However, a significant number (29%, *n* = 9 for image analysis and 35%, *n* = 11 for quality monitoring) expect widespread AI implementation only after 2027, underscoring the potential uncertainty of AI utility among the group, the complexity of implementation, and perhaps the lack of institutional support as these tools require both capital and manpower dedication.

### Conclusions

Excitement due to the potential for AI to improve efficiency and quality in clinical microbiology practice was recognized by the participants of ASM CMO 2024. We recognize that AI is beginning to change how we practice clinical microbiology including quality monitoring, how we find and communicate information using language, and interpretation of visual data (e.g., images of Petri plate and microscopic images). Anticipated or current challenges regarding AI use in clinical practice include the lack of local laboratory expertise in AI development, limited experience in maintaining and adapting algorithms as data evolve over time, and an uncertain regulatory environment.
